# Optimizing knowledge and behavioral intention of women and their partners in the perinatal period in South Africa: a randomized control trial study protocol in the Tshwane district, Gauteng province, South Africa

**DOI:** 10.1186/s12889-022-13600-3

**Published:** 2022-06-20

**Authors:** Elizabeth Leonard, Zwannda Kwinda, Till Baernighausen, Mithilesh Dronavalli, Maya Adam, Yogan Pillay

**Affiliations:** 1 Clinton Health Access Initiative South Africa, 1166 Francis Baard St, Hatfield, Pretoria, 0028 South Africa; 2 Heidelberg University, Grabengasse 1, 69117 Heidelberg, Germany; 3 University of New South Wales, Sydney, NSW 2052 Australia; 4 Stanford University School of Medicine, 291 Campus Drive, Stanford, CA 94305 USA; 5 Department of Global Health, Stellenbosch University, Stellenbosch, 7602 South Africa

**Keywords:** mHealth, Maternal health, Human centered design, Randomized controlled trial, Study protocol, South Africa

## Abstract

**Background:**

Maternal knowledge that motivates improvements in critical perinatal health behaviors has the potential to significantly reduce maternal and neonatal mortality. However, evidence-based health information often fails to reach mothers and their partners. mHealth video micro-messages, which disseminate evidence-based perinatal health messages, have the potential to address this gap.

**Methods:**

The study will make use of a mixed method design, using both qualitative and quantitative methods. The study consists of two phases. During Phase 1, qualitative in-depth interviews will be used as part of a human-centered design approach to co-create 10 videos on priority perinatal behaviors. During Phase 2, quantitative methods (a randomized control trial) will be used to test the effectiveness of the videos in improving maternal knowledge and intended behavioral change.

**Discussion:**

We hypothesize that by engaging mothers and their partners through emotive, resonant narratives and visuals, we can facilitate the delivery of evidence-based health messages at the foundation of perinatal health, thereby motivating life-saving improvements in health behaviors during the perinatal period.

**Trial registration:**

This trial has been prospectively registered on the Pan African Clinical Trials Registry (PACTR), with the registration number PACTR202203673222680. Registration date: 14 March 2022.

**Trial registration WHO data set:**

Registry – Pan African Clinical Trials Registry (PACTR). Date: 14 March 2022. Secondary identification number - grant number: GCCSOAFMNH1. Source of support: Science for Africa Foundation. Primary sponsor – Clinton Health Access Initiative South Africa. Secondary sponsor - Stanford University School of Medicine and Heidelberg University. Contact for public & scientific queries: amandlamamasa@clintonhealthaccess.org; +27 123,426,911; 1166 Francis Baard St, Hatfield, Pretoria, 0028. Public title – Amandla Mama. Scientific title - Optimizing knowledge and behavioral intention of women and their partners in the perinatal period in South Africa. Countries of recruitment – South Africa. Health conditions – antenatal care. Intervention – Amandla Mama mHealth videos, short 2D animated health promotional videos that promote healthy behavior in expectant mothers. Inclusion and exclusion criteria - Expectant mothers and their partners must be 18 years and older. Study type – randomized control trial. Date of first enrollment – 14 March 2021. Sample size – plan to enrol 450 participants, participants enrolled 29 participants. Recruitment Status – suspended. Primary outcome – improving knowledge. Secondary outcome – intended behavioral change. Ethics review – Approved on 24 January 2022 by Pharma-Ethics, contact Mrs. Marzelle Haskins, marzelle@pharma-ethics.co.za. Completion date – N/A. Summary results – N/A. IPD sharing statement – yes, through the publication of results in a journal article.

**Supplementary Information:**

The online version contains supplementary material available at 10.1186/s12889-022-13600-3.

## Background

Maternal knowledge that motivates improvements in critical perinatal health behaviors has the potential to significantly reduce maternal and new-born mortality [1]. However, particularly in the African context, evidence-based health information and key recommendations often fail to reach mothers in formats that are accessible, effective, and compelling.

Catalyzed by increasing access to smartphones and other mobile technologies, mHealth has emerged as a promising proposition for health messaging in countries like South Africa [2] and other low- and middle-income countries [3]. Successful government mHealth initiatives like Mom-Connect, a text-support service for expectant and new mothers, are widely recognized and positively received [4]. However, overcoming literacy barriers and optimizing learner engagement are significant challenges to reaching the broadest possible maternal audience and their partners [5].

Decades of research have underscored the power of using locally resonant narratives to support health behavior change [6]. This has spurred innovations in mHealth video content development [7] that harness the power of, an evidence-based, human-centered design approach. This approach [8] could meaningfully inform the design of “video micro-messages”; which are short 2D animated mHealth videos that enable the cost-effective adaptation and dissemination of evidence-based health information, even in global regions where data costs are high. Adaptable “micro-messages” are small-file, animated short videos that are free of ethnic or socioeconomic identifiers, allowing them to be customized for different audiences by changing only the voiceover, to satisfy language, voice and narrative familiarity.

The South African National Department of Health has been collaborating with health educators at Stanford University and global health researchers at Heidelberg University since 2018, when they began experimenting with the use of simple, animated, story-based health communication tools for the promotion of basic health literacy in the general public [9]. These tools, developed in South Africa by a local production team, incorporated a human-centered design approach, harnessing the power of local voices and local stories to share health information. Today, these early prototypes are being used in South Africa as communication support tools for government-initiated campaigns (like the Road to Health campaign) and by community health workers during their home visits. These video interventions were also subsequently adapted for use (through voiceover swapping and subtle narrative localization) in eSwatini, Burkina Faso, Tanzania, China and Guatemala.

The Amandla Mama study will advance the development and deployment of mHealth communication support interventions. The goal of these interventions is to address existing perinatal knowledge gaps in women and their partners attending public sector services. “Amandla Mama” was chosen as the name for the research study to reflect the study’s goal; “Amandla Mama” translates from Zulu and Xhosa into “Motherly Strength” or “Power to the mother”.

The main aim of this study is to co-create and test the impact of Amandla Mama videos, a series of 10 video-based, narrative, mHealth “micro-messages”, designed to improve knowledge and motivate behavior change. To design the most effective content, we will engage with stakeholders (expectant mothers, partners and health workers) to explore barriers to practicing priority perinatal health behaviors as well as the key messages relevant to priority perinatal topics. Additionally, we will investigate the effect of the Amandla Mama Videos on knowledge and behavioral intentions of women and their partners in the perinatal period.

The specific objectives of the Amandla Mama study are to:

i. Co-create a narrative, video-based mHealth series of 10 “micro messages”.
ii. Assess whether watching the Amandla Mama video series results in a change in knowledge of maternal and neonatal health.
iii. Assess whether watching the Amandla Mama video series changes the intended maternal health behavior.
iv. Assess user satisfaction after engaging with the Amandla Mama video series.

The priority perinatal health behaviors that will be covered in the Amandla Mama Videos are (i) antenatal care and HIV, (ii) birth preparedness, (iii) maternal nutrition, (iv) recognizing danger signs in the new-born period, (v) maternal mental health, (vi) immunizations, (vii) recognizing danger signs in pregnancy, (viii) breastfeeding, (IX) alcohol and drug avoidance, (X) kangaroo mother care / skin-to-skin contact. The study will further explore how universal visuals can be integrated with customized audios to facilitate “glocalization” and finally will test the effectiveness of video micro-messaging for improving maternal knowledge and behavioral intentions as a predictor for behavior change.

## Methods

This study uses a mixed method design, incorporating both qualitative and quantitative methods. The study consists of two phases:

- Phase 1 (qualitative): Qualitative, in-depth interviews will be used as part of a human-centered design approach to create the 10 Amandla Mama videos.
- Phase 2 (quantitative): A randomized control trial will be used to test the effectiveness of the videos.

**Fig. 1:**
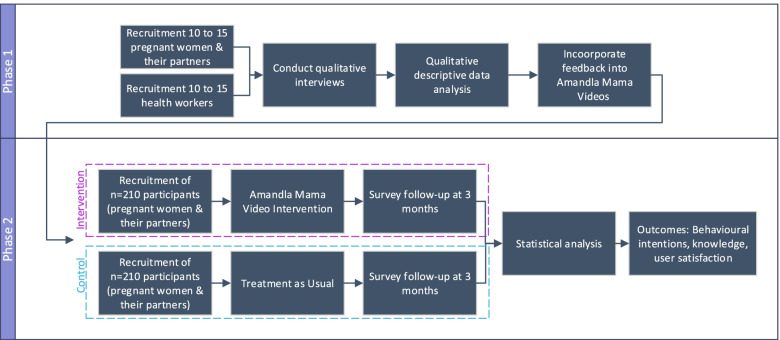
Overview of the Amandla Mama Study Methodology

An overview of the study methodology is shown in Fig. 1.

In Phase 1 the Amandla Mama videos will be iteratively developed through, (i) scripting, (ii) storyboard development, and (iii) refining the developed storyboards by incorporating formative qualitative feedback from expectant mothers, their partners, and health workers gathered through in-depth interviews.

During Phase 2 of the study, the Amandla Mama videos, developed in Phase 1, will be tested using a randomized control trial. Participants will be expectant mothers and their partners living in South Africa accessing routine perinatal healthcare services. The intervention group will watch the Amandla Mama Videos, and the control group will receive treatment as usual for antenatal care only. Treatment as usual antenatal care consists of standard maternity care, as outlined in the South African National Department of Health’s “*Guidelines for Maternity Care in South Africa*” [10]. Maternity care is offered free of charge in the South African public health sector and is integrated into primary healthcare [10]. A survey will be used to gauge whether the intervention results in knowledge change. The surveys, developed by the research team, have been designed to measure change in maternal knowledge, intended behavior, and, user satisfaction. These will be administered to patients 3 months after receiving the intervention. Surveys will also be conducted with partners of the recruited expectant mothers in the study, given the documented benefits to mothers having partners participate in accessing maternal health care services. The partner surveys will have the same content as those designed for the expectant mothers but will be adapted to be relevant to the partner. Knowledge will be the primary outcome and intended behavior change will be the secondary outcome of this randomized control trial.

### Study site

The study sites are two high volume community health centers (CHC) in the Tshwane district of Gauteng province in South Africa, FF Ribeiro Clinic and Kgabo CHC. FF Ribeiro Clinic is situated in the central business district of Tshwane, catering to an urban community and Kgabo CHC, is situated in a large settlement, Ga-Rankuwa, catering to a semi-rural community.

### Study population

The study population includes expectant mothers, 18 years or older attending antenatal care services, pregnant women’s partners, and health workers. Expectant mothers without partners will not be excluded from participating in our study.

The inclusion criteria for expectant mothers and their partners include:

- Expectant mothers and their partners must be 18 years and older.
- Expectant mothers must be accessing antenatal care services at either FF Ribeiro Clinic or Kgabo CHC.
- Expectant mothers and their partners must provide informed consent to participate in the study.

The inclusion criteria for health workers include:

- Health workers must be 18 years and older.
- Health workers must be knowledgeable about antenatal care and work with mothers and their partners.
- Health workers must provide informed consent to participate in the study.

There are no specific exclusion criteria beyond the inclusion criteria.

### Sampling and data collection

To collect valuable qualitative data during Phase 1 of the study, purposive sampling (non-probability sampling) will be utilized. During the quantitative Phase 2, random sampling will be utilized to create an unbiased representation of the study population. The random allocation will be generated by MD. ZK, EL and the research assistants will be responsible for enrolling and assigning participants.

#### Phase 1 sampling & data collection

During Phase 1, purposive sampling will be used to select women and their partners attending antenatal care visits at the study sites for in-depth interviews. Health workers will be purposively selected based on their designated positions. Selected health workers could include community health workers, maternal-child health decision makers or experts, CHC managers, maternity and neonatal unit managers and sub-district or district managers of maternal, neonatal and child health programs. Only participants who provide informed consent to participate in the first phase of the study will be interviewed. The interview guide used during Phase 1 is presented in Additional file 1: Annexure 1.

In-depth interviews will be conducted with 10-15 pregnant women, who present for an antenatal care visit at the study sites. Additionally, in-depth interviews will be conducted with 10-15 health workers at the study sites. A semi-structured questionnaire will be used to guide the qualitative interviews of Phase 1.

The researchers will recruit expectant mothers and their partners after they present at the study sites for an antenatal care visit and will explain the purpose of the study. Expectant mothers who show an interest in participating in the qualitative study will be screened to assess if they meet the inclusion criteria. Informed consent will then be obtained from eligible expectant mothers and their partners. Researchers and research assistants will conduct the interviews in a private area at the respective study sites. All participants will receive ZAR 100 to compensate them for their time and expenses incurred by participating in our study.

#### Phase 2 sampling & data collection

During Phase 2 of the study, the randomized control trial, researchers and research assistants will randomly recruit women attending an antenatal care visit and their partners, inviting them to participate in the randomized control trial. Every participant will be required to provide informed consent before they can be included in the sample. Participants will be randomized into either the intervention group or the control group until the sample size of 420 has been reached. To implement the randomization, a table with the generated randomization sequence will be used, and as a participant agrees to participate in the study, they will be sequentially allocated to a row in the table that will determining the group that the participant will form part of. The sample size for the randomized control trial, was determined using the primary outcome of the study, change in knowledge. We will allocate participants equally to the treatment and control groups, through a balanced computer-generated random sequence. We use a 0.05 level of significance (*P* < 0.05) and a power of 0.9 with a two tailed test for significance.

Our hypothesis is that at the 3-month follow-up, participants in the control group will score 43% on the intention to change behavior score (12 points out of 28 points in the survey). We propose that after the 3-month follow-up the intervention group will score 61% (17 out of 28 points). This is an 18% absolute increase and a 1.41 times relative increase. A sample size of *n* = 140 was calculated for the intervention group and the control group. We then used an increase of 50% to account for potential participant drop-outs, bringing the sample size for the intervention group to *n* = 210 and the sample size for the control group to n = 210, spread equally and randomly across the two sites (Kgabo CHC[urban site] and Ga-Rankuwa[rural site]). Thus, a total of *n* = 420 participants for the entire randomized control trial will be required. These sample size calculations were carried out on G-Power 3.1.9.6.

Sample size calculations were based on the difference between two independent proportions using a z-test (Power: 0.9, Alpha 0.05, Allocation N1/N2: 1, p1 = 0.43, p2 = 0.61). The sample size per group was rounded up to the nearest 10 patients (N1 = 140, N2 = 140).

Expectant mothers assigned to the intervention group will watch the 10 Amandla Mama videos and their partners will receive the same 10 Amandla Mama videos via the social media platform, WhatsApp or short messaging service (SMS). The expectant mothers will be asked to provide the researchers with their contact details and the contact details of their partners. Partners who agree to participate in the study and who provide informed consent will receive the Amandla Mama videos by SMS or WhatsApp. The control group will receive existing treatment as usual-antenatal care only.

After 3 months, knowledge, behavioral intent, and user satisfaction surveys will be administered telephonically to all participants. The follow-up is conducted after 3 months to test long-term retention providing a better predictor for ongoing change. The research assistants under supervision of the researchers, will administer the telephonic survey. Data collected from participants who discontinue with or deviate from the study will not be used.

Surveys for the expectant mothers consist of a total of 61 questions: 10 background questions, 15 behavioral intention questions, 30 knowledge questions and 8 user satisfaction questions (refer to Additional file 1: Annexure 2). Surveys for partners consist of a total of 63 questions: 8 background questions, 18 behavioral intention questions, 30 knowledge questions and 8 user satisfaction questions (refer to Additional file 1: Annexure 3). The user-satisfaction questions, which consider how satisfied the participants are with the Amandla Mama videos, will only be administered to participants in the intervention group. All Phase 2 study participants will receive ZAR 100 to compensate them their time and expenses incurred by participating in our study.

### Data analysis

As a mixed-methods approach will be used for the study, two different data analysis approaches will be used. In Phase 1, a qualitative descriptive approach will be used, and in Phase 2, a statistical analysis will be conducted.

#### Phase 1 data analysis

The qualitative data collected during the Phase 1 interviews will be transcribed, coded in two cycles analyzed using a qualitative descriptive approach. The qualitative descriptive approach has been successfully used to interpret and describe a broad variety of perspectives on health interventions. This naturalistic approach recognizes the subjective nature of participants’ preferences and is often incorporated into mixed-methods studies. This approach also supports the co-creation of health messages that relate to the participants’ local cultures and experiences to optimize engagement and support quality improvements, rather than advancing theoretical or conceptual understanding in this phase [11].

#### Phase 2 data analysis

For Phase 2 of the study, descriptive statistics will be collected and reported to compare the two groups. Confounders that will be measured include demographic characteristics of age, education, race group, religion, nationality, employment and earnings. Another confounder that should be balanced is the exposure of participants to Mom-Connect, an online platform that provides mothers with information about maternal and neonatal care. Confounders will be reported by video intervention and treatment as usual (TAU) across rural and urban sites and overall, for the entire cohort (see mock Table 1). Since this is a large enough randomized control trial the confounders should be balanced. We will compare differences between the control and intervention groups in knowledge and intended behavior change at a 3-month follow-up.

**Table 1:** Mock table of confounders

Socio-Demographic Variables		Urban			Semi-Rural			Overall		
Variable Groupings	Individual Variables/ Questions	Video Intervention	TAU ANC Brochure	*P*-value	Video Intervention	TAU ANC Brochure	P-value	Video Intervention	TAU ANC Brochure	*P*-Value
	Age									
	Standard/grade									
Race	Race 1									
	Race 2									
	Race 3									
Religion	Religion 1									
	Religion 2									
	Religion 3									
Nationality	Nationality 1									
	Nationality 2									
	Nationality 3									
	Employment									
	Earnings									
	Partnership status									

The outcome variables will also be described overall and by rural and urban sties (see mock Table 2). The reported effect size will be an unadjusted relative risk using Poisson regression. Rates for Poisson regression will be total correct answers divided by total questions. This keeps with the intention to treat paradigm, so questions not attempted or answered as “don’t know” are included as incorrect answers).

**Table 2:** Mock table of outcome variables

Variable Groupings	Individual Variables/ Questions	Urban (%)	Semi-Rural (%)	***P***-Value
**Behavioural intentions Mothers**	Attend ANC appointments			
	Own HIV status known			
	Partner HIV status known			
	Take partner to ANC			
	Drink alcohol			
	Wash hands			
	Wear a mask			
	Breastfeed			
	Eat green vegetables			
	Eat fruit			
	Eat meat			
	Take partner to birth			
	Feel fetal kicks			
	Vaccinate child			
	Hold baby			
**Behavioural intentions Partners**	Go to ANC visit			
	Physically enter ANC			
	Pay ANC costs			
	Remind about ANC			
	Ask about ANC			
	Own HIV status known			
	Partners HIV status known			
	Discourage alcohol			
	Encourage wash hands			
	Encourage wear a mask			
	Encourage breastfeeding			
	Provide green vegetables			
	Provide fruit			
	Provide meat			
	Attend birth			
	Encourage fetal kick feel			
	Take vaccination			
	Encourage hold baby			
**Knowledge Score**	Skin to skin KMC			
	Breastmilk prevent infections			
	Breastmilk alone			
	Breastmilk and water			
	Formula feeding			
	Traditional tea/muti			
	Avoid alcohol			
	Source of iron			
	Enough iron			
	Cows milk			
	Postnatal care			
	Condom			
	HIV medication			
	HIV positive breastfeeding			
	Stop breastfeeding			
	Vaccines protect			
	Dangerous vaccines			
	Difficulty sleeping			
	Severe headaches			
	Baby kicks			
	Bleeding			
	Newborn loose weight			
	Newborn fever			
	Cries a lot			
	Black stools			
	Visit clinic			
	Excercise			
	Solid foods			
**User Satisfaction**	Easy to understand			
	Useful			
	Difficult to watch			
	Download easy			
	Download time			
	Follow information			
	Identify with characters			
	All information needed			

Results will be reported by comparing video intervention arm and treatment as usual across rural and urban sites and by outcome sub-groupings (see mock Table 3). Statistical significance will be set at 5% and results will be presented with 95% Confidence Intervals. A Fisher’s exact *P*-value will be reported for all associations.

**Table 3:** Mock table of results reporting

Outcome Analysis	Urban				Semi-rural				Overall			
Variable Groupings:	Video Intervention	TAU ANC Brochure	RR (95%CI)	*P*-value	Video Intervention	TAU ANC Brochure	RR (95%CI)	*P*-value	Video Intervention	TAU ANC Brochure	RR (95%CI)	*P*-Value
Behavioural intentions Mothers												
Behavioural intentions Partners												
Knowledge Score												
User Satisfaction												

Relative Risk calculated using Poisson Regression (rate of correctly answered questions over total questions)

### Ethical considerations

Ethical approval for the study has been granted by the Human Research Ethics Committee at the Pharma-Ethics and by Clinton Health Access Initiative Research Ethics Committee. Additionally, ethics clearance has been obtained from the Tshwane Regional Research Ethics Committee. Written informed consent will be obtained from prospective research participants prior to them partaking in Phase 1 (Additional file 1: Annexure 4) and Phase 2 (Additional file 1: Annexure 5) of the study. Participation is voluntary and participants can withdraw their consent at any time without any threat or punitive measures.

Under South Africa data protection law “Protection of Personal Information Act 2013” the study sites (FF Ribeiro Clinic and Kgabo CHC) and the researchers (investigators, coordinators and study sponsors) will be jointly responsible as ‘controllers’ to ensure that the participants’ information is safeguarded. Data collected during both phases of the study will be de-identified; participants will be assigned unique identifiers (e.g., Participant 1, Participant 2). Data collected during the study will be stored on an encrypted and secure server which will be password protected and only the researchers and study sponsors will have access to it. The data collected will be deleted 2 years after the study has been completed. Dara.

## Discussion

In this study a human centered design approach will be leveraged to design an mHealth intervention that provides evidence-based messaging to expectant mothers and their partners. The study has two distinct phases – the qualitative phase, Phase 1 which focuses on optimizing the Amandla Mama intervention, and the quantitative, Phase 2 for which a randomized control trial will be carried out to establish the effectiveness of the Amandla Mama intervention at changing knowledge and intended behavior. The advantages of using a mixed methods approach, is the obtainment of a more comprehensive set of results, with conclusions that carry a greater weight considering both the human and statistical factors of a study [12].

The study has two potential limitations for wide generalizability. The study is limited to mothers who are 18 years or older and their partners and focuses on two CHCs in Gauteng province limiting generalizability of the results. Despite the potential limitations, the Amandla Mama video innovation stretches conventional approaches by using a narrative, video-based approach that overcomes literacy barriers while facilitating easy adaptation for different cultural groups.

Customization of the visual design and voiceover of content will facilitate “glocalization” of the Amandla Mama videos – adaption of the videos to suit any local context. These videos can be scaled across South Africa, by translating them to all 11 official languages and later across neighboring countries. Successful “glocalization” will showcase the potential of authentic South-to-South collaborations between African nations; and has the potential to inform policy and practice for future social development and health campaigns.

Once the study has concluded, the Amandla Mama videos will be adapted for presentation on open access learning platforms, like Coursera, where they could reach tens of thousands of international families. There is enormous potential in the use of engaging, animated video health content for the creation of lifesaving, evidence-based health messaging that are cost-effective and easily adaptable across cultures. The versatility of these videos, as well as their small file size, results in flexible distribution potential, even in places where data costs are high. Advancement in health literacy from South-to-South collaboration will empower families and communities supporting better health outcomes of all.

## Supplementary Information

**Additional file 1: Annexure 1.** Qualitative interview guide. **Annexure 2.** Quantitative survey – expectant mothers. **Annexure 3.** Quantitative survey – partners. **Annexure 4.** Informed consent form Phase 1. **Annexure 5.** Informed consent form Phase 2.

## Data Availability

Not applicable.

## Abbreviations

CHC: Community health centers
SMS: Short messaging service
TAU: Treatment as usual
ZAR: South African rand
