# Supplementary material for: Optimizing knowledge and behavioral intention of women and their partners in the perinatal period in South Africa: a randomized control trial study protocol in the Tshwane district, Gauteng province, South Africa
Source: BMC Public Health. 2022 Jun 20;22:1224. doi: 10.1186/s12889-022-13600-3 (PMC9208232; doi:10.1186/s12889-022-13600-3)
Supplement: Supplementary file 1 — Additional file 1: Annexure 1. Qualitative interview guide. Annexure 2. Quantitative survey – expectant mothers. Annexure 3. Quantitative survey – partners. Annexure 4. Informed consent form Phase 1. Annexure 5. Informed consent form Phase 2. [file 12889_2022_13600_MOESM1_ESM.pdf]

Annexure 1: Qualitative interview guide

AMANDLA MAMA
Interview Protocol for Qualitative in-depth Interviews

Interviewer name	
Date of interview	
Interview Site	
Start time of the interview	
End time of the interview	

Please remind the participant that you will be recording this interview and ask if it is OK if you start the recording now.

Questions:

1. Please watch the following video about Kangaroo Mother Care: https://youtu.be/iqZkrAhm-Hk
   a. What did you like about the video? What would you change?
   b. How do you feel about the length of the video?
   c. What do you think about using stories to teach people?
   d. Did you learn something from the videos that you find useful?
   e. Any other comments?

   Probe one at a time. Ask clarifying questions as needed.

2. Now, please watch the following video about Breastfeeding: https://youtu.be/yAP_jFAY3Ms
   a. What did you like about the video? What would you change?
   b. How do you feel about the length of the video?
   c. What do you think about the colors used in this video?
   d. Any other comments?

   Probe one at a time. Ask clarifying questions as needed.

3. We have chosen to draw characters and the story rather than using real people. Do you think we should draw any of the following differently?
   a. Characters
   b. Backgrounds/settings
   c. Colours used, if relevant
   d. Using stories to teach vs simply presenting information

   Probe one at a time. Ask clarifying questions as needed.

4. Now, I'd like to switch gears and ask you a bit about what you think are the two (2) most important messages to convey to a pregnant woman on the following topics:
   a. Birth preparedness
   b. Antenatal care
   c. Danger signs in pregnancy
   d. Nutrition in pregnancy

   Probe one at a time. Ask clarifying questions as needed.

5. Is there anything I haven't asked you that you would like to share?

   Thank you so much for making the time to speak with me.

Annexure 2: Quantitative survey – expectant mothers

AMANDLA MAMA

BEHAVIOUR, KNOWLEDGE AND USER SATISFACTION QUESTIONNAIRE FOR MOTHERS

Interviewer name	
Date of interview	
Interview Site	
Start time of the interview	
End time of the interview	

**SECTION ONE: BACKGROUND**		
No	Questions and Filters	Coding Categories
1	How old are you?	Age in years<br>18-24<br>25-34<br>≥35
2	What is the highest standard or grade you have completed at school?	No formal education<br>Primary school<br>Secondary and above
3	With which race group do you identify?	Black/African<br>Coloured<br>Indian<br>White<br>Other:
4	What is your religion, if you have one?	Christian<br>African Traditional<br>Islam<br>Hindu<br>Judaism<br>Buddhist<br>Other<br>None
5	What nationality are you?	South African<br>Other
6	Have you worked to earn money in the last 12 months?	Yes<br>No
7	How much do you earn per month, before tax, including benefits?	Low income (R1-R5000)<br>Middle income (R5001 – R20 000)<br>High income (>R20 000)
8	Do you have a partner?	Yes<br>No

**SECTION TWO: BEHAVIOURAL INTENTIONS**<br>*For each item below, please indicate how likely you are to do these things in the next months.*		
9	Attend your antenatal care appointments at the clinic	Yes<br>No<br>Maybe

10	Know your HIV status?	Yes<br>No<br>Maybe
11	Know the HIV status of your partner?	Yes<br>No<br>Maybe
12	Take your partner with you when you go for your antenatal visits?	Yes<br>No<br>Maybe
13	Drink alcohol	Yes<br>No<br>Maybe
14	Wash your hands before eating a meal	Yes<br>No<br>Maybe
15	Wear a mask to prevent the spread of COVID-19 infection	Yes<br>No<br>Maybe
16	Breastfeed your own baby for the first six months without giving the baby any other food	Yes<br>No<br>Maybe
17	Eat green vegetables on a daily basis	Yes<br>No<br>Maybe
18	Eat fruit on a daily basis	Yes<br>No<br>Maybe
19	Eat meat at least twice a week	Yes<br>No<br>Maybe
20	Ask your partner to accompany you when you are giving birth?	Yes<br>No<br>Maybe
21	Frequently feel if your baby is kicking during your pregnancy	Yes<br>No<br>Maybe
22	Take your child to the clinic for vaccination	Yes<br>No<br>Maybe
23	Hold your baby close to your skin to settle the baby	Yes<br>No<br>Maybe

SECTION 3: KNOWLEDGE SCORE

For each item below, please indicate how you view these statements:

1	Kangaroo mother care should be avoided because it makes breastfeeding difficult	True<br>False<br>Don't know
2	Drinking breastmilk prevents infections in a baby	True<br>False

		Don’t know
3	Breastmilk alone provides enough nutrition until babies are 6 months old	True<br>False<br>Don’t know
4	Babies should be given water or cooled tea in addition to breastmilk when it is hot outside	True<br>False<br>Don’t know
5	Formula feeding is healthier for babies than breastfeeding if mothers can afford it	True<br>False<br>Don’t know
6	Babies under six months should be given muti if recommended by a sangoma	True<br>False<br>Don’t know
7	Pregnant mothers should avoid fruits because it will be too acidic for the baby	True<br>False<br>Don’t know
8	It’s okay to drink alcohol when pregnant because it relaxes the mother	True<br>False<br>Don’t know
9	Iron is found in green vegetables as well as meat	True<br>False<br>Don’t know
10	It is important for pregnant women to get enough iron in their meals	True<br>False<br>Don’t know
11	Cow’s milk is an excellent source of iron	True<br>False<br>Don’t know
12	Cow’s milk is an excellent source of calcium	True<br>False<br>Don’t know
13	Women don’t need to use a condom during pregnancy	True<br>False<br>Don’t’ know
14	Pregnant women that are HIV positive should still take their HIV medication regularly	True<br>False<br>Don’t’ know
15	A woman should not breastfeed her baby if she is HIV positive and on ART	True<br>False<br>Don’t’ know
16	Vaccines help to protect newborn babies from getting sick	True<br>False<br>Don’t know
17	Vaccines are dangerous for newborn babies and should be delayed until the child is older	True<br>False<br>Don’t know
18	Difficulty sleeping is during late pregnancy is a danger sign	True<br>False<br>Don’t know
19	Severe headaches during late pregnancy are danger signs	True<br>False

		Don't know
20	Feeling your baby kick is normal during late pregnancy	True<br>False<br>Don't know
21	Bleeding from the vagina is normal during late pregnancy	True<br>False<br>Don't know
22	When a newborn baby loses some weight in the first week it is a danger sign	True<br>False<br>Don't know
23	When a newborn has a fever in the first week it is a danger sign	True<br>False<br>Don't know
24	When a newborn cries a lot at night, it is a danger sign.	True<br>False<br>Don't know
25	When a newborn has black stools in the first week, it is a danger sign	True<br>False<br>Don't know
26	Pregnant women should visit the clinic regularly, even if they feel fine	True<br>False<br>Don't know
27	Pregnant women should exercise	True<br>False<br>Don't know
28	Newborn babies can be given porridge to help them gain weight and sleep better.	True<br>False<br>Don't know

**SECTION 4: USER SATISFACTION**<br>*For each item below, please indicate how satisfied you were with the videos you saw:*		
1	Was the video easy to understand? (1-5, with 5 **being very easy to understand**)	Scale of 1-5
2	Do you think the video was useful? (1-5, with 5 **being very useful**)	Scale of 1-5
3	How easy was it for you to watch the videos? (1-5, with 5 **being very useful**)	Scale of 1-5
4	How easy was it for you to download the video? (1-5, with 5 **being very easy to download**)	Scale of 1-5
5	How fast was it to download the video? (1-5, with 5 **being very fast to download**)	Scale of 1-5
6	How likely are you to follow the advice offered in the videos? (1-5, with 5 **being very likely to follow the advice**)	Scale of 1-5

7	How easy was it for you to identify with the characters in the animated videos? (1-5, with 5 **being very easy to identify with the characters**)	Scale of 1-5
8	How strongly do you feel that the video gave you important information in a short time? (1-5, with 5 **being I feel very strongly that the video gave me important information in a short time**)	Scale of 1-5

1= disagree, 5=strongly agree

Annexure 3: Quantitative survey – partners

AMANDLA MAMA

BEHAVIOUR, KNOWLEDGE AND USER SATISFACTION QUESTIONNAIRE FOR PARTNERS

Interviewer name	
Date of interview	
Interview Site	
Start time of the interview	
End time of the interview	

**SECTION ONE: BACKGROUND**		
No	Questions and Filters	Coding Categories
1	How old are you?	Age in years<br>18-24<br>25-35<br>≥35
2	What is the highest standard or grade you have completed at school?	No formal education<br>Primary school<br>Secondary and above
3	With which race group do you identify?	Black/African<br>Coloured<br>Indian<br>White<br>Other:
4	What is your religion, if you have one?	Christian<br>African Traditional<br>Islam<br>Hindu<br>Judaism<br>Buddhist<br>Other<br>None
5	What nationality are you?	South African<br>Other

6	Have you worked to earn money in the last 12 months?	Yes<br>No
7	How much do you earn per month, before tax, including benefits?	Low income (R1-R5000)<br>Middle income (R5001 – R20 000)<br>High income (>R20 000)
8	Do you have a partner?	Yes<br>No

**SECTION TWO: BEHAVIOURAL INTENTIONS**<br>*For each item below, please indicate if you are likely to do these things in the next months:*		
8	Accompany your partner to at least one ANC visit?	Yes<br>No<br>Maybe
9	Physically enter the ANC room together with your partner?	Yes<br>No<br>Maybe
10	Contribute to covering the costs of the partners' ANC visits?	Yes<br>No<br>Maybe
11	Remind your partner about her ANC follow-up visit?	Yes<br>No<br>Maybe
12	Take time to find out what went on during the ANC visits?	Yes<br>No<br>Maybe
13	Know your HIV status?	Yes<br>No<br>Maybe
14	Know your partner's HIV status?	Yes<br>No<br>Maybe
15	Discourage your partner from using alcohol during her pregnancy?	Yes<br>No<br>Maybe
16	Wash your hand before a meal and encourage your partner to wash hand before meals?	Yes<br>No<br>Maybe
17	Wear a mask to prevent the spread of COVID-19 infection and encourage your partner to do the same?	Yes<br>No<br>Maybe
18	Encourage your partner to breastfeed her baby for the first six months without giving the baby any other food?	Yes<br>No<br>Maybe
19	Help to provide your partner with green vegetables to eat on a daily basis?	Yes<br>No<br>Maybe
20	Help to provide your partner with fruit to eat on a daily basis?	Yes<br>No<br>Maybe

21	Help to provide your partner with meat to eat at least twice a week?	Yes<br>No<br>Maybe
22	Accompany your partner when she gives birth in a hospital?	Yes<br>No<br>Maybe
23	Encourage your partner to frequently feel if the baby is kicking during her pregnancy?	Yes<br>No<br>Maybe
24	Take your child to the clinic for vaccination?	Yes<br>No<br>Maybe
25	Encourage your partner to hold the baby close to her skin immediately after the baby is born	Yes<br>No<br>Maybe

**SECTION 3: KNOWLEDGE SCORE**<br>*For each item below, please indicate how you view these statements:*		
1	Kangaroo mother care should be avoided because it makes breastfeeding difficult	True<br>False<br>Don’t know
2	Drinking breastmilk prevents infections in a baby	True<br>False<br>Don’t know
3	Breastmilk alone provides enough nutrition until babies are 6 months old	True<br>False<br>Don’t know
4	Babies should be given water or cooled tea in addition to breastmilk when it is hot outside	True<br>False<br>Don’t know
5	Formula feeding is healthier for babies than breastfeeding if mothers can afford it	True<br>False<br>Don’t know
6	Babies under six months should be given muti if recommended by a sangoma	True<br>False<br>Don’t know
7	Pregnant mothers should avoid fruits because it will be too acidic for the baby	True<br>False<br>Don’t know
8	It’s okay to drink alcohol when pregnant because it relaxes the mother	True<br>False<br>Don’t know
9	Iron is found in green vegetables as well as meat	True<br>False<br>Don’t know
10	It is important for pregnant women to get enough iron in their meals	True<br>False<br>Don’t know
11	Cow’s milk is an excellent source of iron	True

		False<br>Don’t know
12	Cow’s milk is an excellent source of calcium	True<br>False<br>Don’t know
13	Women don’t need to use a condom during pregnancy	True<br>False<br>Don’t’ know
14	Pregnant women that are HIV positive should still take their HIV medication regularly	True<br>False<br>Don’t’ know
15	A woman should not breastfeed her baby if she is HIV positive and on ART	True<br>False<br>Don’t’ know
16	Vaccines help to protect newborn babies from getting sick	True<br>False<br>Don’t know
17	Vaccines are dangerous for newborn babies and should be delayed until the child is older	True<br>False<br>Don’t know
18	Difficulty sleeping is during late pregnancy is a danger sign	True<br>False<br>Don’t know
19	Severe headaches during late pregnancy are danger signs	True<br>False<br>Don’t know
20	Feeling your baby kick is normal during late pregnancy	True<br>False<br>Don’t know
21	Bleeding from the vagina is normal during late pregnancy	True<br>False<br>Don’t know
22	When a newborn baby loses some weight in the first week it is a danger sign	True<br>False<br>Don’t know
23	When a newborn has a fever in the first week it is a danger sign	True<br>False<br>Don’t know
24	When a newborn cries a lot at night, it is a danger sign.	True<br>False<br>Don’t know
25	When a newborn has black stools in the first week, it is a danger sign	True<br>False<br>Don’t know
26	Pregnant women should visit the clinic regularly, even if they feel fine	True<br>False<br>Don’t know
27	Pregnant women should exercise	True<br>False<br>Don’t know

28	Newborn babies can be given porridge to help them gain weight and sleep better.	True<br>False<br>Don't know

**SECTION 4: USER SATISFACTION**<br>*For each item below, please indicate how satisfied you were with the videos you saw:*		
1	Was the video easy to understand? (1-5, with 5 **being very easy to understand**)	Scale of 1-5
2	Do you think the video was useful? (1-5, with 5 **being very useful**)	Scale of 1-5
3	How easy was it for you to watch the videos? (1-5, with 5 **being very useful**)	Scale of 1-5
4	How easy was it for you to download the video? (1-5, with 5 **being very easy to download**)	Scale of 1-5
5	How fast was it to download the video? (1-5, with 5 **being very fast to download**)	Scale of 1-5
6	How likely are you to follow the advice offered in the videos? (1-5, with 5 **being very likely to follow the advice**)	Scale of 1-5
7	How easy was it for you to identify with the characters in the animated videos? (1-5, with 5 **being very easy to identify with the characters**)	Scale of 1-5
8	How strongly do you feel that the video gave you important information in a short time? (1-5, with 5 **being I feel very strongly that the video gave me important information in a short time**)	Scale of 1-5

1= disagree, 5=strongly agree

Annexure 4: Informed consent form Phase 1

AMANDLA MAMA

PARTICIPANT'S INFORMATION LEAFLET AND INFORMED CONSENT

(Each participant must receive, read or have this document read to him/her before the interview)

Phase 1: Qualitative interviews

Qualitative in-depth interviews with maternal-child health stakeholders for storyboard development

Introduction

Good day. My name is _________________. I am working with the non-governmental organization, the Clinton Health Access Initiative, on a research study. We want to develop short video messages to provide mothers and partners with information on caring for themselves and their babies. We are working together with Stanford University in the United States of America and Heidelberg University in Germany on this project.

You are invited to volunteer to participate in a research study that is conducted at two facilities in the Tshwane municipality in the Gauteng Province: FF Ribeiro Clinic in the inner city and Kgabo CHC in Ga-Rankuwa.

You should not agree to participate in this study unless you fully understand what is asked of you and are completely happy with all the procedures involved. If you do not understand the information or have any other questions, please feel free to ask the interviewer for more information or clarification.

Purpose of the study and your participation

The purpose of this study is to develop and test short video messages to support mothers and their partners during pregnancy and after the delivery of the baby. Data for this study will be collected in two phases. In the first phase of the project, we will capture the views on the short video messages from key maternal-child stakeholders (community health care workers, maternal-child health decision makers/experts, facility managers of community clinics and health centres, managers of maternity and neonatal units of hospitals and sub-district and district managers of maternal, neonatal and child health programmes) to include in the development of the final series. The second phase of the study is a randomised control trial where mothers and their

partners will be randomised in an intervention and control group. The intervention group will receive the final series of video messages. Data will be collected at three months follow up using a structured questionnaire.

You have been invited to participate in the first phase of the study because you are an important stakeholder in maternal-child health, and we would like to understand your preferences on these short videos to help mothers during and after pregnancy. We are speaking to a number of stakeholders, such as mothers, their partners, community health care workers and managers in maternal-child health, who have kindly volunteered their time to help us design this collection short videos that will be appealing and helpful to mothers in many different settings. Because the mothers using these videos will be different from one another - and will be living in different parts of the country - we have kept the characters and settings simple. This also helps to keep data costs down because live footage is expensive to send and receive. Our goal is to achieve a style of content that is enjoyable for all mothers to watch - and content that will help them learn more about their health and the health of their families.

Please note that we will extend another invitation for you to participate in the second phase of our study. You will be required to provide another informed consent to participate in the second phase of the study.

What procedures are involved?

With your permission, I will be recording this interview so that we can transcribe it later. The recording itself will not be used for any other purposes, it will be stored securely on a password protected, encrypted computer, and the recording will be deleted after it has been transcribed. This means that your name will not be linked to the answers that you give.

We would like to ask some information about you and then we would like you to watch prototypes of two short micro-messages. We will then have an interview with you on your views on the individual videos. The videos and the interview will take approximately 30 minutes. Please answer truthfully and don't feel you need to answer with what you think we want to hear! There are no right or wrong answers.

Are there any risks or discomforts from participating in this study?

There are minimal potential risks from participating in this study. The videos may evoke unexpected memories and distressing experiences. You may feel uncomfortable answering some questions. There is potential risk of breach of confidentiality in an event of unexpected problem which is/may cause harm to the participant and others.

Possible Benefits of this study

There are no direct benefits that you may get from participating in this study. However, the information collected from this study may be helpful in improving the care of mothers and babies in South Africa. Your answers will help us make sure that the information we received from women and their partners are taken into account in designing future programmes using short video messages.

What are your rights as a participant?

Your participation in this study is entirely voluntary. You can refuse to participate or stop at any time without giving any reason. Your withdrawal from our study will not in any way affect the care you receive from the clinic. Know that your participation in this first phase does not obligate you to participate in the second phase of our study.

Confidentiality

All the information that you give in this study will be kept strictly confidential. The consent forms that you will be asked to sign will be securely stored and access will be limited to the research team and study sponsors. The consent forms cannot be linked to the answers you give to the questionnaire. The results of the study will be presented in a respectful manner and no information which could enable anyone to identify you personally will be reported. If you would like to be kept informed of the progress of our project, we will be happy to share any reports or publication we produce with you.

Under South Africa data protection law “Protection of Personal Information Act 2013” your study site and the sponsor will be jointly responsible as ‘controllers’ to ensure that your information is safeguarded. Your data might be transferred to a country that may not have the same level or personal data protection as South Africa. If your data is transferred outside South Africa the sponsor is responsible for protecting your data.

Costs

There is no cost to you for participating in this study.

Compensation

You will be given R100 to compensate you for the time, expenses, and inconveniences related to your participation in our study.

Has this study received ethical approval?

Yes, this study has received ethical approval from the Pharma-Ethics Independent Research Ethics Committee. The study has also received ethics clearance from the Gauteng Health Research Committee (PHRC).

Information and contact person

If you have any questions or queries about the research, you may contact our Project Coordinator Zwannda Kwinda at [redacted] or email her at [redacted]@clintonhealthaccess.org. You can phone the Pharma-ethics Health Research Ethics Committee at [redacted] or email them at [redacted]@pharma-ethics.co.za. if there still is something that your study doctor has not explained to you, or if you have a complaint. You will receive a copy of this information and consent form for you to keep safe.

Declaration by participant

By signing below, I ……………………………….………… agree to take part in a research study entitled (insert title of study here).
I declare that:

- I have read this information and consent form, or it was read to me, and it is written in a language in which I am fluent and with which I am comfortable.
- I have had a chance to ask questions and I am satisfied that all my questions have been answered.
- I understand that taking part in this study is voluntary, and I have not been pressurised to take part.
- I may choose to leave the study at any time and nothing bad will come of it – I will not be penalised or prejudiced in any way.
- I may be asked to leave the study before it has finished, if the study doctor or researcher feels it is in my best interests, or if I do not follow the study plan that we have agreed on.

Signed at (*place*) ....…........……………….. on (*date*) …………...……….. 2022

____________________ Signature of participant

____________________ Signature of witness

Declaration by investigator
I *(name)* ……………………………………………..……… declare that:

- I explained the information in this document in a simple and clear manner to …………………………………..
- I encouraged him/her to ask questions and took enough time to answer them.

- I am satisfied that he/she completely understands all aspects of the research, as discussed above.
- I did/did not use an interpreter. (*If an interpreter is used then the interpreter must sign the declaration below.)*

Signed at (*place*) ....….......…………….. on (*date*) …………....……….. 2022.

_____________________ _____________________

Signature of investigator Signature of witness

Permission to have all anonymous data shared with journals:

Please carefully read the statements below (or have them read to you) and think about your choice. No matter what you decide, it will not affect whether you can be in the research study, or your routine health care

When this study is finished, we would like to publish results of the study in journals. Most journals require us to share your anonymous data with them before they publish the results. Therefore, we would like to obtain your permission to have your anonymous data shared with journals.

To protect your privacy, we will replace your name with a unique study number. We will only use this code for your sample and information about you. We will do our best to keep the code private. It is however always possible that someone could find out about your name but this is very unlikely to happen. Therefore, we would like to ask for your permission to share your samples and information with other investigators.

Tick the option you choose for anonymous data sharing with journals:

I agree to have my anonymous data shared with journals during publication of results of this study

☐ Signature____________

OR

I do not agree to have my anonymous data shared with journals during publication of results of this study

☐ Signature____________

Annexure 5: Informed consent form Phase 2

AMANDLA MAMA

PARTICIPANT'S INFORMATION LEAFLET AND INFORMED CONSENT
(Each participant must receive, read or have this document read to him/her before the interview)

Phase 2: Randomised Control Trial
Questionnaire on behavioural intention and knowledge of women and their partners before and after birth (perinatal period)

Introduction

Good day. My name is _________________. I am working with the non-governmental organization, the Clinton Health Access Initiative, on a research study. We want to develop short video messages to provide mothers and partners with information on caring for themselves and their babies. We are working together with Stanford University in the United States of America and Heidelberg University in Germany on this project.

You are invited to volunteer to participate in a research study that is conducted at two facilities in the Tshwane municipality in the Gauteng Province: FF Ribeiro Clinic in the inner city and Kgabo CHC in Ga-Rankuwa.

You should not agree to participate in this study unless you fully understand what is asked of you and are completely happy with all the procedures involved. If you do not understand the information or have any other questions, please feel free to ask the interviewer for more information or clarification.

Purpose of the study and your participation

The purpose of this study is to develop and test short video messages to support mothers and their partners during pregnancy and after the delivery of the baby. Data for this study will be collected in two phases. In the first phase of the project, we will capture the views on the short video messages from key maternal-child stakeholders (community health care workers, maternal-child health decision makers/experts, facility managers of community clinics and health centres, managers of maternity and neonatal units of hospitals and sub-district and district managers of maternal, neonatal and child health programmes) to include in the development of the final series. The second phase of the study is a randomised control trial where mothers and their

partners will be randomised in an intervention and control group. The intervention group will receive the final series of video messages. Data will be collected at three months follow up using a structured questionnaire.

You have been invited to participate in the second phase of the study because you were randomly picked/picked by chance during your first antenatal care visit, or because you are a partner to a mother. You are assigned either to a group that will watch short videos or to a group that will not watch any short videos. If you were assigned to the group that will watch the short videos, we want to ask your permission to show you these video messages. After 3 months we would like you to complete a questionnaire which a Research Assistant will administer.

What procedures are involved?

We will ask you to watch 10 short video messages if you were selected by chance. If you are a mother, we will show you these video messages after your antenatal care visit. If you are a partner, we will share these messages with you on WhatsApp or through the SMS messaging system. You will only be able to watch the videos once you have consented to participate in the study, either by signing this form or consenting on WhatsApp or on SMS.

This study will not at all compromise your opportunity to participate in MomConnect, which is a government programme that provides all mothers, who have access to WhatsApp, with information on the health of mothers and babies.

After 3 months, we will call all the participants telephonically – the group that watched the short video-messages and the group that did not watch these messages - to complete a questionnaire. We will call you on the number(s) you provide, and a research assistant will administer the questionnaire telephonically. The questionnaire will consist of a series of questions that will be asking for information about your behaviours, attitude and knowledge of caretaking practices for mothers and babies. It will take approximately 30 minutes to watch the video messages and about an hour to administer the questionnaire three months from the day you watch the videos.

We will ask you to answer truthfully and to remember that there are no right or wrong answers. All your answers will be saved in a computer and your name will not be recorded. We will save the responses that you give to the questionnaire using a unique identification number. This means that your name will not be linked to the answers that you give. Information that is collected from you will be put together with information from 420 other women and their partners.

Are there any risks or discomforts from participating in this study?

There are minimal potential risk from participating in this study. The videos may evoke unexpected memories and distressing experiences. You may feel uncomfortable answering some questions. Certain questions may trigger traumatic experiences. There is potential risk of breach of confidentiality in an event of unexpected problem which is/may cause harm to the participant and others.

Possible Benefits of this study

There are no direct benefits that you may get from participating in this study. However, the information collected from this study may be helpful in improving the care of mothers and babies in South Africa. Your answers will help us make sure that the information we received from women and their partners are taken into account in designing future programmes using short video messages.

What are your rights as a participant?

Your participation in this study is entirely voluntary. You can refuse to participate or stop at any time without giving any reason. Please remember that you are free to skip over any question you do not want to answer, and you are free to stop answering questions at any time. Please note that your withdrawal from our study will not in any way affect the care you receive from the clinic. You are not obligated to participate in this second phase if you were a participant in the first phase of our study.

Confidentiality

All the information that you give in this study will be kept strictly confidential. The consent forms that you will be asked to sign will be securely stored and access will be limited to the research team and study sponsors. The consent forms cannot be linked to the answers you give to the questionnaire. The results of the study will be presented in a respectful manner and no information which could enable anyone to identify you personally will be reported. If you would like to be kept informed of the progress of our project, we will be happy to share any reports or publications we produce with you.

Under South Africa data protection law "Protection of Personal Information Act 2013" your study site and the sponsor will be jointly responsible as 'controllers' to ensure that your information is safeguarded. Your data might be transferred to a country that may not have the same level or personal data protection as South Africa. If your data is transferred outside South Africa the sponsor is responsible for protecting your data.

Costs

There is no cost to you for participating in this study.

Compensation

You will be given R100 to compensate you for the time, expenses, and inconveniences related to your participation in our study.

Has this study received ethical approval?

Yes, this study has received ethical approval from the Pharma-Ethics Independent Research Ethics Committee. The study has also received ethics clearance from the Gauteng Health Research Committee (PHRC).

Information and contact person

If you have any questions or queries about the research you may contact our Project Coordinator Zwannda Kwinda at [redacted] or email her at [redacted]@clintonhealthaccess.org. You can phone the Pharma-ethics Health Research Ethics Committee at [redacted] or email them at [redacted]@pharma-ethics.co.za. If there still is something that your study doctor has not explained to you, or if you have a complaint. You will receive a copy of this information and consent form for you to keep safe.

Declaration by participant

By signing below, I …………………………………..………… agree to take part in a research study entitled (insert title of study here).

I declare that:

- I have read this information and consent form, or it was read to me, and it is written in a language in which I am fluent and with which I am comfortable.
- I have had a chance to ask questions and I am satisfied that all my questions have been answered.
- I understand that taking part in this study is voluntary, and I have not been pressurised to take part.
- I may choose to leave the study at any time and nothing bad will come of it – I will not be penalised or prejudiced in any way.
- I may be asked to leave the study before it has finished, if the study doctor or researcher feels it is in my best interests, or if I do not follow the study plan that we have agreed on.

Signed at (*place*) ....…........……………. on (*date*) …………...……….. 2022

____________________ _________________________

Signature of participant **Signature of witness**

Declaration by investigator

I *(name)* ……………………………………………..……… declare that:

- I explained the information in this document in a simple and clear manner to …………………………………..

- I encouraged him/her to ask questions and took enough time to answer them.
- I am satisfied that he/she completely understands all aspects of the research, as discussed above.
- I did/did not use an interpreter. (*If an interpreter is used then the interpreter must sign the declaration below.)*

Signed at (*place*) ....…........……………….. on (*date*) …………....……….. 2022.

____________________ ____________________

Signature of investigator **Signature of witness**

Permission to have all anonymous data shared with journals:

Please carefully read the statements below (or have them read to you) and think *about your choice. No matter what you decide, it will not affect whether you can be in the research study, or your routine health care*

When this study is finished, we would like to publish results of the study in journals. Most journals require us to share your anonymous data with them before they publish the results. Therefore, we would like to obtain your permission to have your anonymous data shared with journals.

To protect your privacy, we will replace your name with a unique study number. We will only use this code for your sample and information about you. We will do our best to keep the code private. It is however always possible that someone could find out about your name but this is very unlikely to happen. Therefore, we would like to ask for your permission to share your samples and information with other investigators.

Tick the Option you choose for anonymous data sharing with journals:

I agree to have my anonymous data shared with journals during publication of results of this study

☐ **Signature**____________

OR

I do not agree to have my anonymous data shared with journals during publication of results of this study

☐ **Signature**____________
